# Utilization of spectral filteration for ultra‐low dose brain CT in pediatric patients for diagnosis of craniosynostosis: A phantom study

**DOI:** 10.1002/acm2.70176

**Published:** 2025-07-14

**Authors:** Connor Braniff, Michael Ditchfield, Manuel Gubser, Ahilan Kuganesan, M. K. Badawy

**Affiliations:** ^1^ Royal Melbourne Institute of Technology Melbourne Victoria Australia; ^2^ Monash Health Imaging Monash Health Clayton Victoria Australia; ^3^ Department of Paediatrics Monash University Melbourne Australia; ^4^ Division of Radiology and Nuclear Medicine St. Gallen Cantonal Hospital St. Gallen Switzerland; ^5^ Department of Medical Imaging and Radiation Sciences School of Primary and Allied Health Care Faculty of Medicine Nursing and Health Sciences Monash University Clayton Victoria Australia

**Keywords:** craniosynostosis, low‐dose computer tomography, pediatric, SilverBeam, tin filter

## Abstract

**Objectives:**

This study evaluates the effectiveness of spectral filtration—specifically Tin and SilverBeam filters—in achieving ultra‐low radiation doses in pediatric computed tomography (CT) imaging for craniosynostosis diagnosis. We investigate whether these filters can reduce radiation to levels comparable to or below those of standard four‐view skull x‐rays, while maintaining diagnostic accuracy. Unlike previous research focused broadly on dose reduction, this study highlights the potential of Tin and SilverBeam filtration as a promising solution.

**Methods:**

CT images were acquired using a pediatric head fracture phantom, with sutures simulating craniosynostosis, on two scanners with different spectral filters. The CTDIvol was reduced by varying percentages from standard pediatric protocols using Tin and SilverBeam filters. Image quality and radiation dose were quantitatively assessed, while two radiologists performed qualitative evaluations.

**Results:**

Dose comparisons showed that images with at least a 89.2% reduction in CTDIvol using the Tin filter and a 91.4% reduction when using the SilverBeam filter resulted in lower doses than a standard four‐view skull x‐ray (0.09 mSv). The greatest dose reduction (up to 161%) occurred with SilverBeam at a 99.9% reduction in CTDIvol. While significant pixel intensity changes were observed at the sutures, spatial resolution decreased at lower dose settings. Two pediatric radiologists observed clear skull and orbit outlines under clinical conditions, but coronal sutures became undetectable near 90% CTDIvol reduction.

**Conclusion:**

Spectral filtration ultra‐low‐dose CT significantly reduces radiation exposure compared to four‐view skull x‐rays, showing promise for diagnosing craniosynostosis. Further patient‐based studies are needed to validate diagnostic efficacy.

**Advances in knowledge:**

This study is the first to assess spectral filtration in ultra‐low‐dose pediatric head CT, highlighting significant dose reductions without compromising diagnostic quality, and stressing the need for further validation.

## 1 INTRODUCTION

Craniosynostosis is a condition characterised by the premature closure of the fibrous sutures in the skull, which restricts normal brain and skull growth and potentially increases intracranial pressure. Often associated with genetic syndromes, it frequently necessitates surgical intervention.^1^, ^2^ The primary diagnostic tool for craniosynostosis in pediatric patients is the four‐view skull x‐ray, which captures multiple angles of the skull but presents challenges, particularly with the submentovertex (SMV) view, as it requires tilting the neck, causing patient discomfort and distress. Furthermore, the need for multiple exposures heightens the risk of motion artefacts, compromising diagnostic quality.^3^ CT offers a promising alternative by capturing all views in a single scan, being faster, reducing discomfort, and minimizing artefacts. However, concerns about higher radiation doses, especially in children, limit its use. Children's increased sensitivity to radiation and longer life expectancy make them more susceptible to radiation‐induced harm.^4^, ^5^ For CT to be a viable alternative, it must deliver doses comparable to, or lower than, four‐view skull x‐rays or provide greater diagnostic confidence. Spectral filters, such as Tin and Silver, offer a potential solution by attenuating lower‐energy photons, thereby decreasing radiation exposure, although there can be an impact on image quality.^6^

Several studies have explored low‐dose CT protocols for the diagnosis of pediatric craniosynostosis. Barreto et al.^7^ and Lyoo et al.^8^ investigated ultra‐low‐dose CT protocols combined with advanced reconstruction techniques, such as deep learning, to reduce radiation exposure. Barreto's study achieved a 98% reduction in radiation dose compared to conventional pediatric CT protocols, lowering the dose to levels comparable with four‐view skull x‐rays. Lyoo's research focused on integrating ultra‐low‐dose CT with deep learning reconstruction, demonstrating that diagnostic accuracy could be maintained despite the substantial dose reduction. Although image quality was initially inferior to routine dose protocols, applying deep learning reconstruction preserved diagnostic performance. These findings suggest that deep learning reconstruction, combined with low‐dose CT protocols, could be an effective alternative to traditional radiography for diagnosing craniosynostosis.^7^, ^8^

While previous research has explored dose reduction in pediatric CT for craniosynostosis, recent advancements in spectral filters such as Tin and SilverBeam remain underexplored—particularly when combined with advanced reconstruction methods. These filters were selected for evaluation due to their recent introduction in clinical practise and limited prior study in this clinical context. Our study evaluates this combined approach, integrating spectral filtration with deep learning reconstruction (AiCE, Canon) and iterative reconstruction (ADMIRE, Siemens), to determine whether it can achieve radiation doses lower than those of four‐view skull x‐rays while maintaining diagnostic image quality in a phantom model.

## 2 METHOD

### 2.1 Study design

This study employed an experimental design using a pediatric whole‐body fracture phantom (PBU‐70B, representing a five‐year‐old child; Kyoto Kagaku, Kyoto, Japan) to simulate the anatomical structures of pediatric patients. While the phantom does not replicate the pathological features of craniosynostosis, its anatomically accurate head shape and visible sutures serve as a reasonable surrogate for assessing image quality and spatial resolution relevant to cranial structure visualization. Although suture visibility in a healthy phantom cannot be directly extrapolated to diseased states, this approach enables a controlled evaluation of the detectability of fine cranial features under varying low‐dose CT acquisition parameters, allowing us to assess the limits of image quality and spatial resolution in detecting features relevant to craniosynostosis diagnosis, in a phantom model without patient‐related variability or radiation exposure from experimental protocols.

New data were collected exclusively for this study, and no human participants were involved; therefore, ethical approval was unnecessary. The study was conducted within a single health network using two CT scanners: a Siemens SOMATOM Force (Siemens Healthineers AG, Forchheim, Germany) and a Canon Aquilion One Prism (Canon Medical, Ōtawara, Japan).

### 2.2 Imaging protocol

CT scans of the head phantom were performed on both Siemens and Canon CT scanners, using identical setup and scan ranges to ensure consistency. Each scanner used its respective standard pediatric head protocol, with the Canon Aquilion One Prism protocol serving as the reference for baseline image quality in this study. This protocol was selected as the standard due to its higher acquisition parameters, typically associated with improved image quality. This Canon‐based protocol will be referred to as the “paediatric standard” throughout the paper and was also used for all qualitative image analyses. (Table 1).

After the reference scan, additional scans were performed using either the SilverBeam or Tin spectral filter. These filters are not permanently installed but can be selectively activated via filter‐specific protocols available on their respective CT platforms. Tin‐filter scans were performed at 100 kVp, reducing the CTDIvol by 72.4%, 89.2%, 94.6%, and 96.4% relative to the Siemens Somatom Force standard pediatric protocol (Table 2). SilverBeam scans, performed at 120 kVp, achieved reductions in CTDIvol of 91.4%, 99.5%, 99.7%, 99.8%, and 99.9% from the Canon Acquilion One pediatric protocol (Table 2).

**TABLE 1:** Pediatric protocol for Siemens and Canon Scanners.

Acquisition	kVp	mA	CTDIvol (mGy)	DLP (mGy.cm)	Pitch	Rotation	Reconstruction algorithm
Siemens SOMATOM Force	100	613	7.60	124.3	0.5	0.5	ADMIRE (Hr59h)
Canon Aquilion One	120	50	9.30	135.40	0.63	0.5	AiCE (Bone BHC)

**TABLE 2:** Silverbeam and Tin Filter acquisition parameters.

Filter	Reduction in CTDIvol(%)	kVp	mA	CTDIvol (mGy)	DLP (mGy.cm)	Pitch	Rotation time	Reconstruction algorithm
Tin Filter (Sn)	96.4	100	19	0.27	4.50	0.5	0.5	ADMIRE (Hr59h)
	94.6		30	0.41	6.70			
	89.2		60	0.82	13.4			
	72.4		152	2.10	34.2			
SilverBeam filter (Ag)	99.9	120	10	0.10	2.40	1	0.5	AiCE (Bone BHC)
	99.8		15	0.20	3.60			
	99.7		25	0.30	6.00			
	99.5		50	0.50	12.00			
	91.4		80	0.80	19.20			

The 99.8% reduction (SilverBeam) and 96.4% reduction (Tin filter) in CTDIvol represent the lowest achievable tube current settings for each filter, defining the minimum dose limits evaluated in this study. Additionally, higher‐dose settings were selected to assess how image quality degrades as CTDIvol decreases, allowing us to identify the lowest acquisition parameters that still preserve diagnostic utility for each filter. All dose reductions are reported as percentage decreases in CTDIvol relative to the corresponding standard pediatric protocols.

### 2.3 Dose estimates

A retrospective audit was conducted on 20 randomly selected patients with craniosynostosis who had undergone four‐view skull x‐rays, serving as a reference for dose comparison. Data were collected between November 2023 and June 2024 using the Radiology Information System (RIS, Philips, version 11.5.0.5), employing an exact text search for “craniosynostosis” with the procedure labeled “XR Skull X‐ray.” The Picture Archiving and Communication System (PACS, Philips, V.12.2.6) was then utilised to analyse the relevant DICOM data for the dose area product (DAP) and peak kilovoltage (kVp).

Radiographic x‐ray dose estimates were performed using NCIRF v.2.0.2, incorporating the average DAP values for each projection angle from the four‐view skull audit. The primary and secondary angles of the positioner were adjusted to correspond with the Towne's, Lateral, Anterior‐Posterior, and SMV views. Calculations utilized an energy of 60 kVp and a half‐value layer (HVL) of 2.25 mm, running simulations for 100 000 histories across 24 threads. The effective doses in millisieverts (mSv) were then aggregated to provide a reference dose comparison for the four‐view skull x‐ray.^10^ The effective dose for each low‐dose CT acquisition was calculated in millisieverts by multiplying the dose‐length product of each acquisition by a conversion factor of 0.004 mSv/mGy/cm, corresponding to the age profile of the pediatric head CT of the PBU‐70B 5‐year‐old phantom.^9^, ^10^

### 2.4 Image analysis

Images produced under various acquisition settings were analysed both quantitatively and qualitatively to assess image quality and diagnostic confidence. Quantitative analysis involved the use of line profiles to evaluate pixel intensity changes at the sutures, while full width at half maximum (FWHM) was used to assess spatial resolution for axial slices, where all sutures were visible.

To evaluate suture visibility in this phantom study, we adopted a conservative threshold of 120 pixel intensity units as a marker of visual discernibility. These pixel intensities were analyzed from raw DICOM data, correlating with respective changes in Hounsfield Units (HU). This threshold was used to determine whether the contrast between a suture and the surrounding bone was sufficient for detection under typical diagnostic conditions. A change of ≥120 units in pixel intensity between the background and the suture was considered a conservative threshold for visual discernibility, serving as a reproducible marker for identifying structures that would likely be visible under routine diagnostic conditions.

This threshold corresponds to an approximate 5%–6% contrast change across the visible greyscale range and is consistent with values reported in contrast‐detail phantom evaluations and observer detection studies.^11^, ^12^ Although DICOM images store up to 256 greyscale levels (8 bits per pixel), the human eye can typically distinguish only 60–80 levels simultaneously under optimal viewing conditions. In clinical CT imaging, the minimum detectable difference in soft tissue has been estimated at approximately 4–6 Hounsfield Units (HU) for moderately sized objects under low‐noise conditions^12^ (ACR CT Accreditation Guidelines). Detectability also depends on additional factors such as display window width, image noise, anatomical background, and object size. While smaller HU differences may be perceptible in idealised scenarios, the 120‐pixel intensity threshold was selected as a conservative and reproducible estimate of contrast detectability that remains valid across a range of acquisition parameters in this phantom study.^11^, ^12^, ^13^

Inter‐rater agreement among the radiologists was assessed using Cohen's Kappa to evaluate consistency in identifying suture visibility based on the 120‐pixel intensity threshold, allowing for assessment of agreement between their gradings.

**FIGURE 1:**
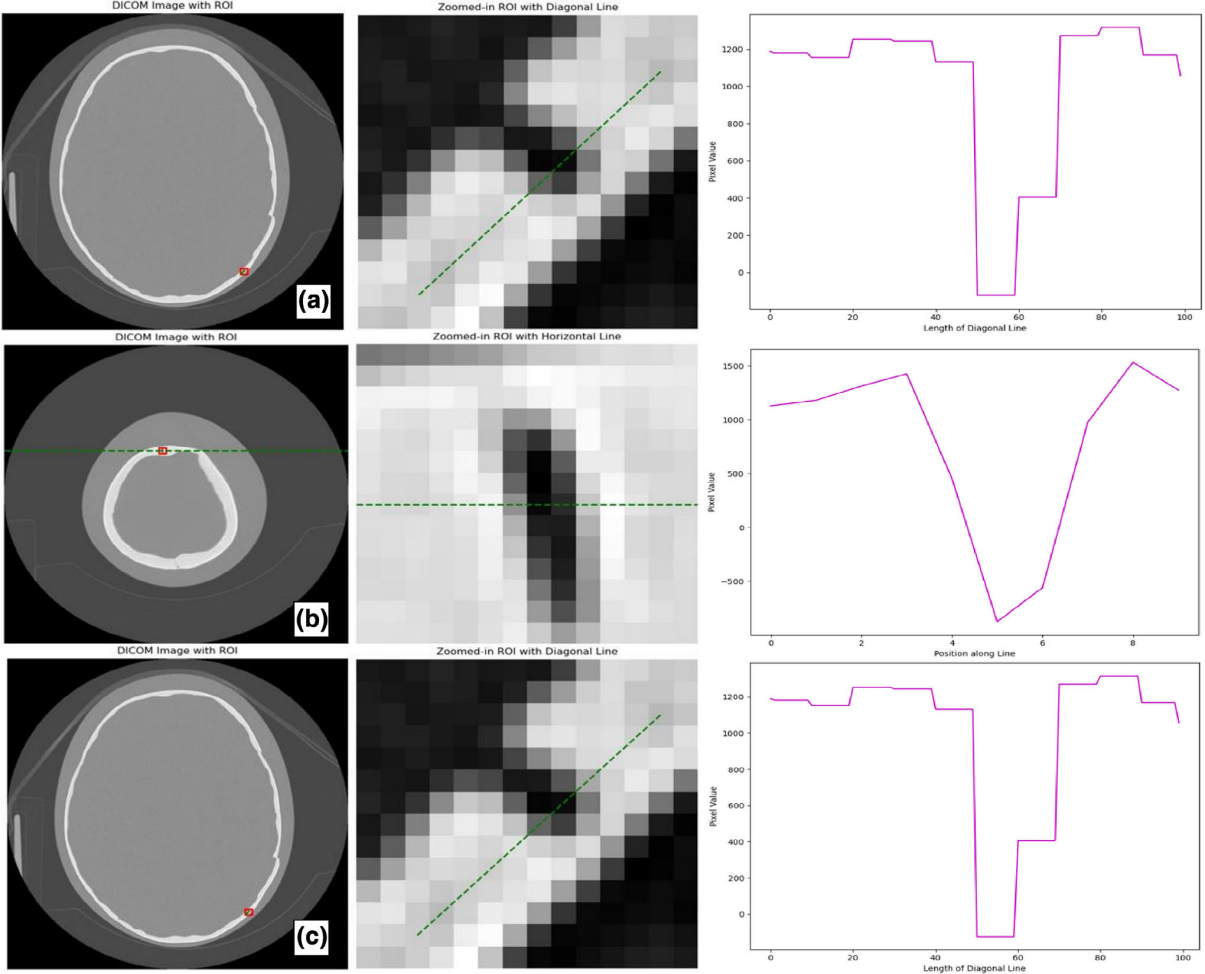
Line profile fitting for (a) Lambdoid, (b) Sagittal, and (c) coronal sutures, utilizing a standard pediatric CT protocol for the Canon Aquilion One. The acquisition parameters are detailed in Table 1, enabling the examination of pixel variations associated with the presence of each suture.

Figure 1 illustrates the line profiles taken in regions of interest (ROIs) for the right Lambdoid, the Sagittal, and right Coronal sutures using the standard pediatric head CT protocol. The ROIs for all three sutures were selected for each acquisition to generate figures analysing pixel intensity changes and FWHM. FWHM analysis for each line profile helped assess image resolution. A narrower FWHM indicates higher spatial resolution and improved clarity of anatomical details, whereas a broader FWHM signifies decreased spatial resolution and reduced image clarity.^14^

Lastly, a qualitative analysis was conducted by two pediatric radiologists without prior access to annotations. All images obtained utilizing the Tin and Silverbeam filter were reviewed, as well as those taken under pediatric protocol with the Canon Aquilion One, which served as the diagnostic standard. The radiologists were asked to score all images as seen, partially seen or not seen, based on the visibility of the skull and orbit shape, as well as the clarity of sutures to allow the determination of the lowest acquisition parameters possible before diagnostic confidence is lost.

## 3 RESULTS

**FIGURE 2:**
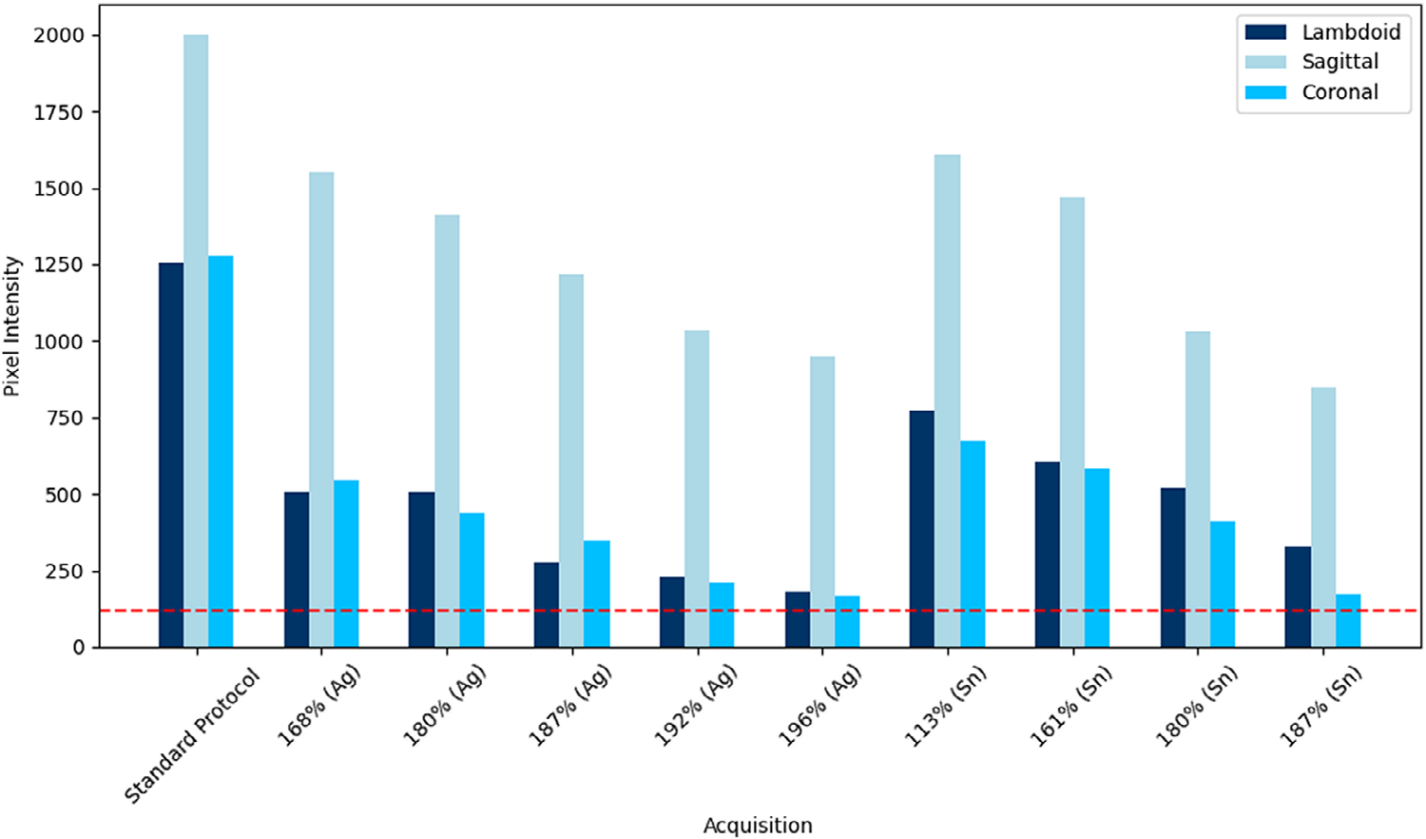
Changes in pixel intensity associated with a suture across all filter acquisitions, as specified in Table 2. (The red line represents a 120‐pixel value threshold, below which the density change related to the presence of the suture is no longer visible to the human eye.)

Figure 2 illustrates the pixel intensity changes across all acquisitions and respective sutures, highlighting that all sutures exhibited a pixel intensity change of greater than 120. This level of contrast suggests that sutures are likely visually discernible to the human eye.

**FIGURE 3:**
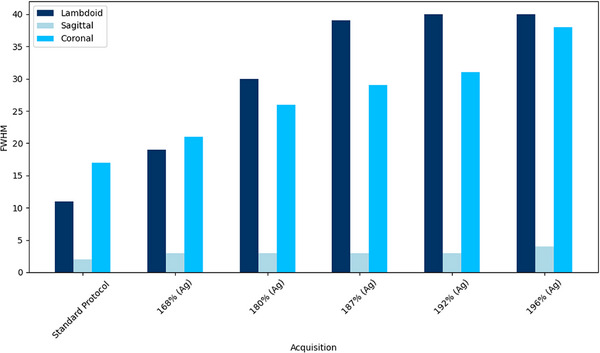
FWHM for the coronal lambdoid, and sagittal sutures across Silverbeam acquisitions. A reduction in FWHM signifies a decline in spatial resolution.

**FIGURE 4:**
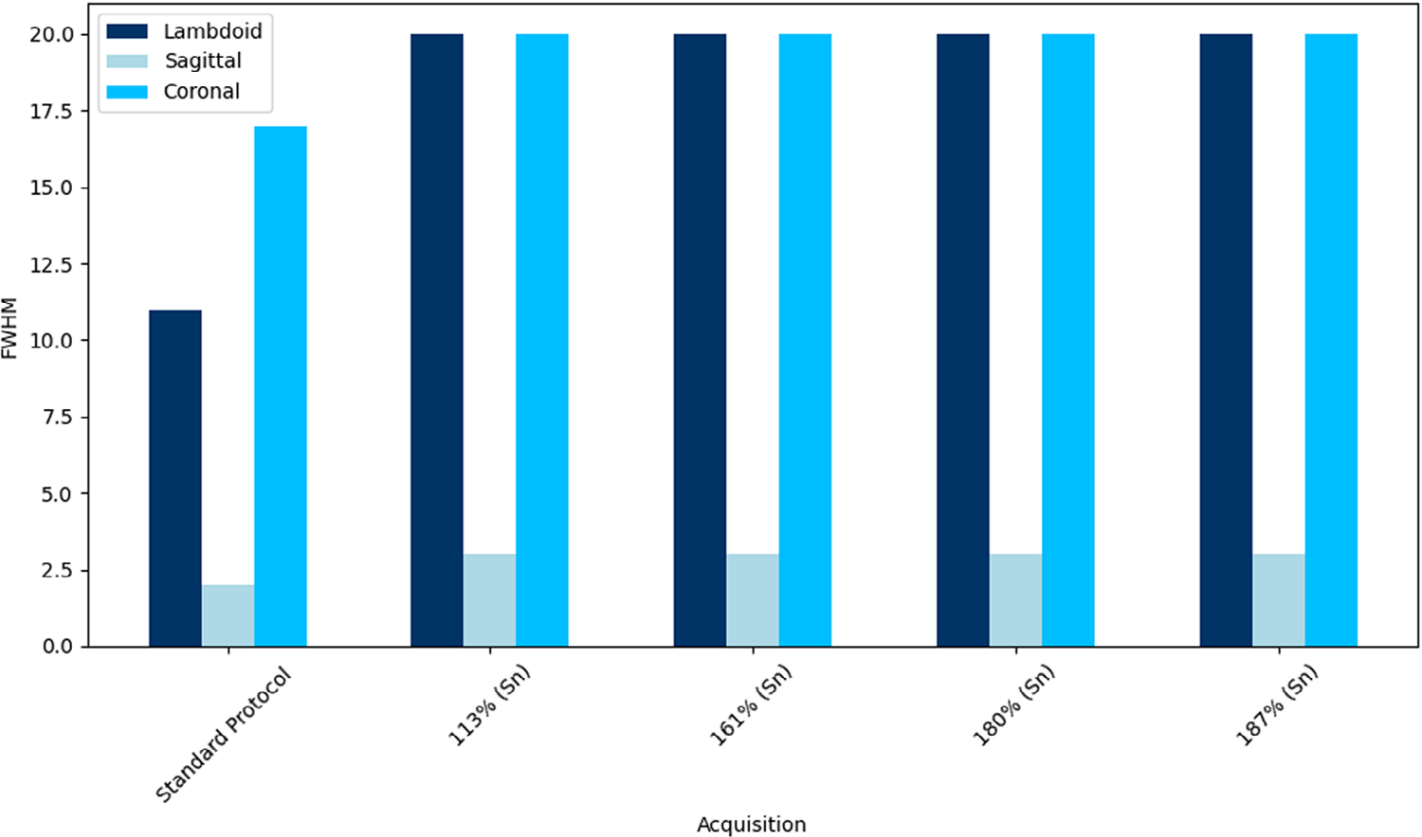
FWHM for coronal, lambdoid, and sagittal sutures over tin filter acquisitions. A reduction in FWHM signifies a decline in spatial resolution.

Figures 3 and 4 display the FWHM values for the SilverBeam and Tin filters, respectively. The SilverBeam shows an increase in FWHM as CTDIvol values decrease, indicating a loss in spatial resolution. In contrast, the FWHM for Tin filters remains constant across all acquisitions for a particular suture, suggesting that spatial resolution is maintained despite the lowering of acquisition parameters.

The radiologists' assessment, as summarized in Table 3, indicates that the shape of the skull and orbit was discernible in all acquisitions, facilitating the identification of any abnormal growth. The Sagittal and Lambdoid sutures were at least partially visible across all acquisition parameters. However, the Coronal sutures were not visible in acquisitions below a decrease in CTDIvol of 91.4% or 89.2% using the Silverbeam and Tin filters, respectively. Cohen Kappa scores indicated high to moderate agreement in scoring between radiologists for the Sagittal and Lambdoid sutures, with low agreement for the Coronal suture.

**TABLE 3:** Radiologist scoring of anatomical visibility under various CT acquisition settings, including filter types, CTDI reduction percentage, across suture and skull regions (coronal, sagittal, lambdoid, skull, and orbit).

Filter type	Reduction in CTDIvol (%)	Coronal right		Coronal left		Sagittal		Lambdoid right		Lambdoid left		Skull shape	Orbit shape
Tin (Sn)	96.4	×	±	×	±	±	±	±	√	±	√	Yes	Yes
	94.6	×	±	×	±	√	±	√	√	√	√	Yes	Yes
	89.2	±	±	±	±	√	√	√	√	√	√	Yes	Yes
	72.4	±	√	±	√	√	√	√	√	√	√	Yes	Yes
SilverBeam (Ag)	99.9	×	×	×	×	±	±	±	±	±	±	Yes	Yes
	99.8	×	×	×	×	±	±	±	±	±	±	Yes	Yes
	99.7	×	×	×	±	±	±	±	±	±	±	Yes	Yes
	99.5	×	×	×	±	±	±	±	±	±	±	Yes	Yes
	91.4	±	±	±	±	±	√	±	±	±	±	Yes	Yes
Pediatric Standard	N/A	±	√	±	√	√	√	√	√	√	√	Yes	Yes
Cohen Kappa Scores	N/A	0.44		0.21		0.60		0.80		0.80		1.0	1.0

Symbols for radiologist scoring: Seen (√), Partially Seen (±), and Not Seen (×).

Table 4 presents the calculated radiation doses for all CT acquisition settings and the four‐view skull radiograph. The standard acquisition had a dose‐length product (DLP) of 135.4 mGy·cm and an effective dose of 0.54 mSv. Acquisitions using the SilverBeam filter demonstrated effective dose reductions between 91.4% and 99.9%. Acquisitions using the Tin filter showed effective dose reduction between 89.2% and 96.4%.

**TABLE 4:** Dose calculation for the four‐view skull x‐ray, the standard pediatric protocol, and all acquisitions utilizing Silverbeam and Tin filters.

Filter	Acquisition settings	DLP (mGy.cm)	Effective dose (mSv)	% Difference from 4‐view skull x‐ray
N/A	Standard pediatric protocol	135.4	0.55	143
N/A	Four‐view skull x‐ray	N/A	0.10	N/A
SilverBeam (Ag)	99.9%	2.4	0.009	—162
	99.8%	3.6	0.01	—145
	99.7%	6	0.02	—116
	99.5%	12	0.05	—61
	91.4%	19.2	0.08	—16
Tin Filter (Sn)	96.4%	4.5	0.02	—145
	94.6%	6.7	0.03	—108
	89.2%	13.4	0.05	—51
	72.4%	34.2	0.14	41

The effective dose for the spectral acquisitions was calculated by multiplying the dose‐length product of each acquisition by a conversion factor of 0.004 mSv/mGy/cm, corresponding to the pediatric head CT for a 5‐year‐old phantom.

## 4 DISCUSSION

The line profile analysis revealed that all sutures demonstrated intensity differences exceeding 120 pixel values across all filter acquisitions, suggesting they should be visually discernible under standard viewing conditions. However, as shown in Figure 2, the intensity difference decreased progressively with reduced dose, with some acquisitions approaching the 120‐pixel threshold. This trend indicates that lowering acquisition parameters compromises the contrast between bone and suture, potentially affecting visual detectability.

Furthermore, the increase in FWHM at lower CTDIvol settings, particularly with the SilverBeam filter, reflects a reduction in spatial resolution. This reduction compounds the visibility challenge, especially for finer structures such as the coronal sutures, which were rated as “Not Seen” by both radiologists at the lowest acquisition levels (see Table 4). While the pixel intensity threshold provides a useful reference point, visibility is not solely determined by this factor.

It is essential to emphasize that the 120‐pixel threshold is a simplification based on average human greyscale perception and does not fully capture the complexity of detectability. In clinical imaging, detectability is influenced by several contextual variables, including noise level, contrast‐to‐noise ratio (CNR), viewing conditions, and anatomical background texture. Observer variability further complicates the threshold at which differences become perceptible. As such, while the 120‐pixel value served as a reproducible and conservative marker in this study, it should not be interpreted as a universal threshold. Further research involving patient data, observer studies, or model observers would be necessary to refine this estimate for clinical application; however, this was beyond the scope of the present phantom‐based study.

As illustrated in Figure 2, the Sagittal suture exhibits the slightest change in pixel intensity and FWHM across varying acquisition parameters. The FWHM remains relatively low, even at the lowest dose values, indicating good spatial resolution at reduced exposure levels. This superior spatial resolution observed in the Sagittal suture is likely attributable to its larger width compared to other sutures in the images produced, resulting in a more substantial area of black pixels corresponding to the absence of bone and the presence of the suture. The wider sagittal suture width is expected, as supported by L.A. Mitchell,^15^ with the Sagittal suture being almost double the width of the Coronal suture for pediatric patients aged 0–12 months. This broader gap in the sagittal suture creates more explicit boundaries and more distinct features, with greater pixel representation capturing the suture's presence. Consequently, the Sagittal suture maintains good spatial resolution even at lower exposure settings.^15^

Figure 4 shows that FWHM remains consistent across acquisition settings when using the Tin filter, indicating that the iterative reconstruction algorithm effectively preserves spatial resolution despite reduced dose. In contrast, Figure 3 demonstrates that the FWHM for SilverBeam and AiCE reconstructions increases with decreasing CTDIvol values, indicating a decline in spatial resolution under these conditions. However, although FWHM remains stable with the Tin filter, radiologists in Table 3 reported a reduction in suture detectability as the CTDIvol was reduced. This suggests that lowering CTDIvol affects image quality in ways not fully captured by FWHM alone, likely due to the increased image noise at lower dose levels, which can impair the perceptual visibility of fine structures.

Furthermore, the comparison of effective doses reveals that a standard pediatric brain CT scan delivers an effective dose approximately six times higher than the four‐view skull x‐ray, with the CT scan registering an effective dose of 0.54 mSv for the PBU‐70B phantom compared to 0.09 mSv from the four view skull the x‐ray dose audit. This substantial difference emphasises that, although CT scans may enhance patient comfort by reducing the need for multiple exposures, they expose pediatric patients to significantly higher levels of ionising radiation. Consequently, dose reduction is imperative to make CT scans a viable imaging option for diagnosing craniosynostosis without compromising patient safety.

Table 4 outlines the reductions in radiation dose achieved by each acquisition compared to the standard four‐view skull x‐ray. To achieve dose reductions, a minimum decrease of 91.4% in CTDIvol was required for the Silverbeam filter and 89.2% decrease for the Tin filter, resulting in dose reductions of 50.7% and 15.8%, respectively, with all sutures assessed as at least “partially visible” under these acquisitions. The most significant dose reductions were recorded at an 99.9% and 96.4% reduction in CTDIvol, resulting in dose reductions of 161.5% (0.009 mSv) and 144.8% (0.02 mSv) for the Silverbeam filter and the Tin Filter, respectively. These values are lower than the estimated effective dose reported in similar studies, such as those by Barreto et al.,^7^ Lyoo et al.,^8^ and Montoya et al.,^16^ who reported effective doses of 0.06, 0.05, and 0.04 mSv, respectively. However, both radiologists scored the left and right coronal sutures as “Not Seen” at acquisitions taken below reduction in CTDIvol of 91.4% for the SilverBeam and 89.2% for the Tin filter, indicating that these lower acquisition parameters may yield images with limited diagnostic value.^16^

The radiologists' findings indicate that neither the left nor right coronal sutures are visible in images when acquisition parameters are reduced below a 91.4% decrease in CTDIvol for the SilverBeam and a 89.2% reduction in the Tin filter. However, this does not necessarily imply that lower acquisition parameters are unsuitable for diagnosing craniosynostosis. A key limitation of the phantom study is that the phantom's head does not exhibit craniosynostosis, making it difficult to definitively assess the diagnostic confidence for this condition, even at higher acquisition settings. This limitation prevents us from conclusively determining diagnostic efficacy based solely on suture visibility and orbit/skull shape. Nevertheless, using SilverBeam with a 91.4% decrease in CTDIvol and a Tin filter with a 89.2% reduction in CTDIvol still resulted in a reduced dose compared to a four‐view skull x‐ray, while achieving at least partial visibility of all sutures. However, this does not account for potential ridge formation in fused sutures, which might be detectable at lower acquisition parameters.

Several limitations must be acknowledged. First, as a phantom‐based study, our findings cannot directly assess diagnostic efficacy for craniosynostosis. The phantom used does not exhibit pathological features such as suture fusion or ridge formation, which are critical for clinical diagnosis. Second, three‐dimensional reconstruction capabilities—routinely used in clinical practice to enhance the visualisation of skull morphology and sutures—were not evaluated in this study. Their inclusion could potentially improve diagnostic accuracy and permit further dose reductions, and increase the widespread applicability of results. Third, no additional observer performance testing, such as receiver operating characteristic (ROC) analysis, was conducted. This omission limits our ability to assess how radiologists might interpret these images in a real‐world setting. However, Cohen's Kappa was used to assess inter‐rater agreement, highlighting moderate to substantial agreement between radiologists for the scoring of Sagittal (0.6) and Lambdoid Sutures (0.8), while only fair agreement was observed for the Coronal (0.44). Conducting further performance testing would have provided a more comprehensive understanding of diagnostic utility in clinical practice. These limitations highlight the need for future patient‐based trials to validate diagnostic performance, optimise acquisition parameters, and determine the clinical utility of low‐dose CT protocols incorporating spectral filters and advanced reconstruction algorithms.

## 5 CONCLUSION

This study demonstrates that the use of spectral filters—specifically SilverBeam and Tin—in combination with deep learning and iterative reconstruction algorithms can enable substantial reductions in radiation dose for pediatric CT imaging. While this phantom‐based work does not directly assess diagnostic accuracy for craniosynostosis, it demonstrates that key anatomical features relevant to diagnosis, such as skull shape and suture visibility, can be imaged at doses lower than those of traditional four‐view skull x‐rays. These findings support the potential for clinical application but underscore the need for future clinical studies to validate diagnostic performance, optimize acquisition protocols, and confirm clinical utility under real‐world conditions.

## AUTHOR CONTRIBUTIONS

All authors contributed to the study conception and design. C. Braniff and M. K. Badawy completed material preparation, data collection and quantitative analysis. A. Kuganesan contributed to data acquisition. M. Ditchfield and M. Gubser made significant contribution to qualitative analysis. All authors read and approved the final manuscript.

## CONFLICT OF INTEREST STATEMENT

The authors have no relevant conflicts of interest to disclose.

## Data Availability

Available upon request, contact corresponding author.

## References

[1] Sharma R . Craniosynostosis. Indian J Plastic Surg. 2013;46(1):18. 10.4103/0970-0358.113702 PMC3745117 23960302

[2] Faasse M , Mathijssen IMJ . Guideline on treatment and management of craniosynostosis: patient and family version. J Craniofac Surg. 2023;34(1):418‐433. ERN CRANIO Working Group on Craniosynostosis. 36472893 10.1097/SCS.0000000000009143 PMC9794150

[3] Sadrameli M , Mupparapu M . Oral and maxillofacial anatomy. Radiol Clin N Am. 2018;56(1):13‐29. 29157543 10.1016/j.rcl.2017.08.002

[4] Kutanzi K , Lumen A , Koturbash I , Miousse I . Pediatric exposures to ionizing radiation: carcinogenic considerations. Int J Environ Res Public Health. 2016;13(11):1057. 10.3390/ijerph13111057 27801855 PMC5129267

[5] ‘CT Scans for Children: information for Referrers’. Australian Radiation Protection and Nuclear Safety Agency; 2015.

[6] Sandborg M , Carlsson CA , Carlsson GA . Shaping X‐ray spectra with filters in X‐ray diagnostics. Med Biol Eng Comput. 1994;32:384‐390. doi:10.1007/BF02524689 7967802

[7] Barreto IL , Tuna IS , Rajderkar DA , Ching JA , Governale LS . Pediatric craniosynostosis computed tomography: an institutional experience in reducing radiation dose while maintaining diagnostic image quality. Pediatr Radiol. 2022;52(1):85‐96. 10.1007/s00247-021-05205-6 34731286

[8] Lyoo Y , Choi YH , Lee SB . Ultra‐low‐dose computed tomography with deep learning reconstruction for craniosynostosis at radiation doses comparable to skull radiographs: a pilot study. Pediatr Radiol. 2023;53(11):2260‐2268. 10.1007/s00247-023-05717-3 37488451

[9] NCICT. ′NCI dosimetry system for computed tomography. National Cancer Institute; 2021. doi:10.1088/2057-1976/acd2de

[10] McCollough C , Cody D , Edyvean S , et al. 2008. ‘The Measurement, Reporting, and Management of Radiation Dose in CT’, AAPM. 10.37206/97

[11] Kimpe T , Tuytschaever T . Increasing the Number of Gray Shades in Medical Display Systems—How Much is Enough?. Journal of Digital Imaging. 2007;20: 422‐432. doi:10.1007/s10278-006-1052-3 17195900 PMC3043920

[12] Alsleem HA , Almohiy HM . The Feasibility of Contrast‐to‐Noise Ratio on Measurements to Evaluate CT Image Quality in Terms of Low‐Contrast Detailed Detectability. Med Sci. 2020;8(3):26. doi: 10.3390/medsci8030026 PMC7563972 32640553

[13] America College of Radiology. ‘ACR CT Accreditation Guidelines’. American College of Radiology; 2024.

[14] Alshweikh AM , Kusminarto K , Suparta GB . An Improved Method of Measuring Spatial Resolution of the Computed Tomography from ESF based on CT phantom images. Int J Appl Eng Res. 2018. https://www.ripublication.com/ijaer18/ijaerv13n15_86.pdf

[15] Mitchell LA , Kitley CA , Armitage TL , Krasnokutsky MV , Rooks VJ . Normal Sagittal and Coronal Suture Widths by Using CT imaging. Am J Neuroradiol. 2011;32(10):1801‐1805. doi:10.3174/ajnr.A2673 21920859 PMC7966025

[16] Montoya JC , Eckel LJ , DeLone DR , et al. Low‐dose CT for craniosynostosis: preserving diagnostic benefit with substantial radiation dose reduction. Am J Neuroradiol. 2017;38(4):672‐677. 28183836 10.3174/ajnr.A5063 PMC7960256

